# Supplementary material for: A graphical pipeline platform for MRS data processing and analysis: MRSpecLAB
Source: Front Neuroimaging. 2025 Jul 18;4:1610658. doi: 10.3389/fnimg.2025.1610658 (PMC12313582; doi:10.3389/fnimg.2025.1610658)
Supplement: Supplementary file 1 [file Data_Sheet_1.DOCX]

Supplementary Material

# **Supplementary Section 1.** **Overview of MRS/MRSI pre-/processing and quantification tools**

Comparison of commonly used MRS/MRSI software tools. The table summarizes each tool’s primary functions, programming language, availability of graphical user interfaces (GUI), licensing model, support for compiled versions, and feature coverage including MRSI, X-nuclei, edited MRS, and compatibility with NIfTI (.nii) format.

MRSpecLAB stands out for its integrated user-friendly pipeline builder, comprehensive modality support, and open-source Python-based implementation with compiled executables.


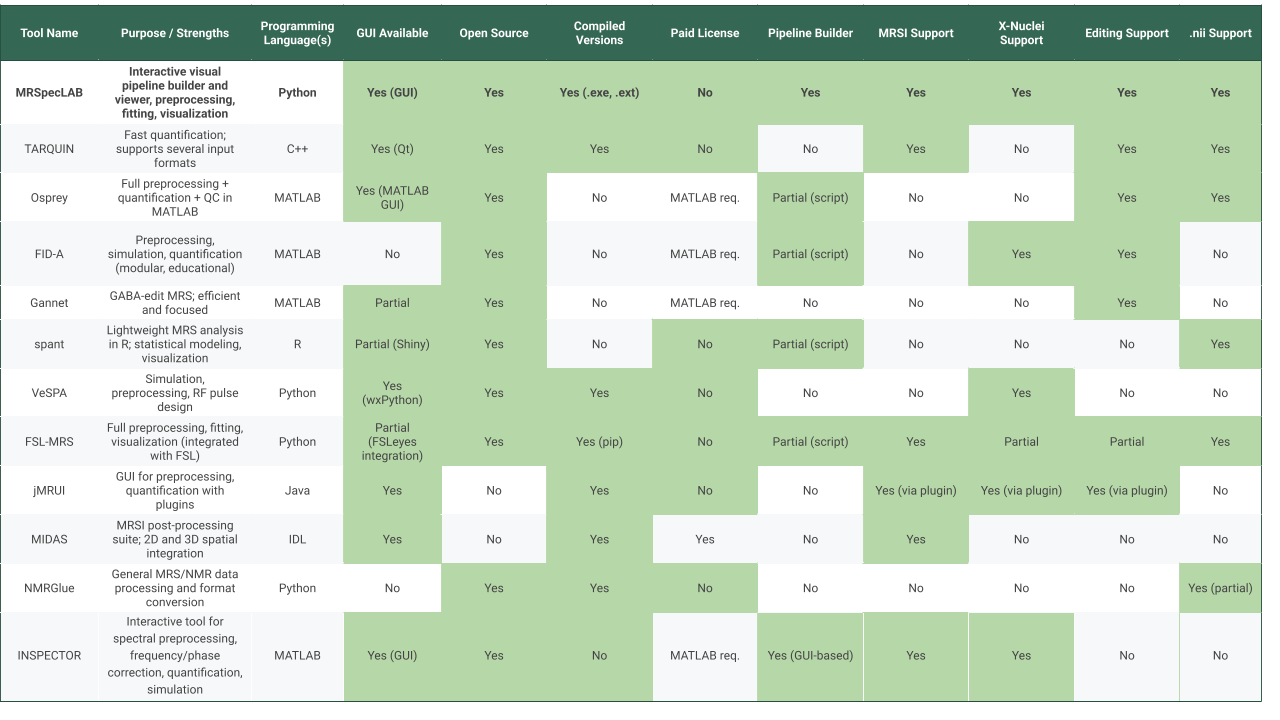


# **Supplementary Section 2. Step-by-step processing and visual outputs of short-TE ^1^H data**

A series of screenshots illustrating each processing step for another short-TE ^1^H dataset, which focuses on processing and quantifying short-TE ^1^H dataset, which acquired from the human brain on Siemens Terra.X 7T scanner with 1TX / 32RX Head Coil (Nova Medical). The example dataset were both in Siemens raw data format (.dat), included water-suppressed data acquired using the STEAM sequence (TE/TR = 4.5/4000 ms, bandwidth = 4 kHz, voxel size = 30 × 30 × 30 mm^3^, 32 averages) and unsuppressed water data obtained using the same sequence and parameters, from the human parietal lobe.

The screenshots demonstrate how MRSpecLAB performs essential processing steps with the intuitive interface and the default-setting pipeline, such as (1) adaptive coil combination, (2) frequency and phase alignment, (3) eddy current correction, (4) bad average removal, (5) quality matrix, (6) spectral averaging, and (7) spectral fitting with LCModel. The results, including figures of processed data, fitting diagnostics, and quantification outputs, were generated and stored automatically.

1. Adaptive coil combination: The input raw data for this step consists of uncombined signals from 32 individual receiver channels. During the adaptive coil combination process, MRSpecLAB automatically combines these signals based on the input unsuppressed water data. The algorithm takes into account variations in coil sensitivities and phases across channels, applying phase correction and weighting to achieve the optimal combination. In the central plot panel, the data visualization of this step is organized into two rows. The first row displays the time-domain data, allowing users to observe the raw signal and its changes throughout this step. The second row showcases the frequency-domain data, presenting the spectra before and after variation corrections, which provides a clear, side-by-side comparison of the data before and after combination.

**
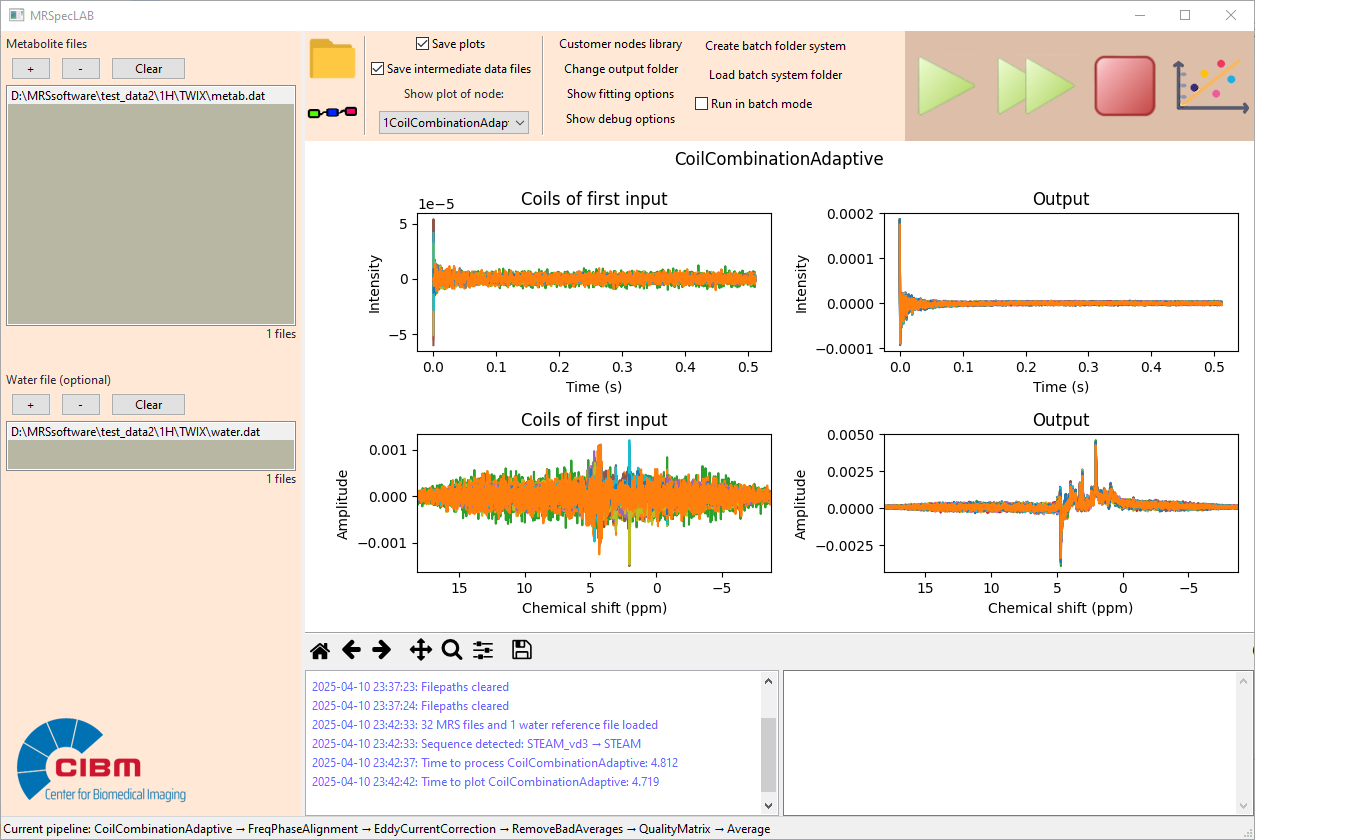
**

1. Frequency and phase alignment: The data after coil combination proceeds to the alignment of individual transients utilizing the N-acetylaspartate (NAA) peak at 2.02 ppm as the reference. Three-fold zero padding was applied to the input data, followed by broadening using a Lorentzian function with a linewidth of 5 Hz. These preprocessing steps, illustrated in the left figure of the second row, were performed to enable accurate estimation of frequency and phase drifts in the dataset.

**
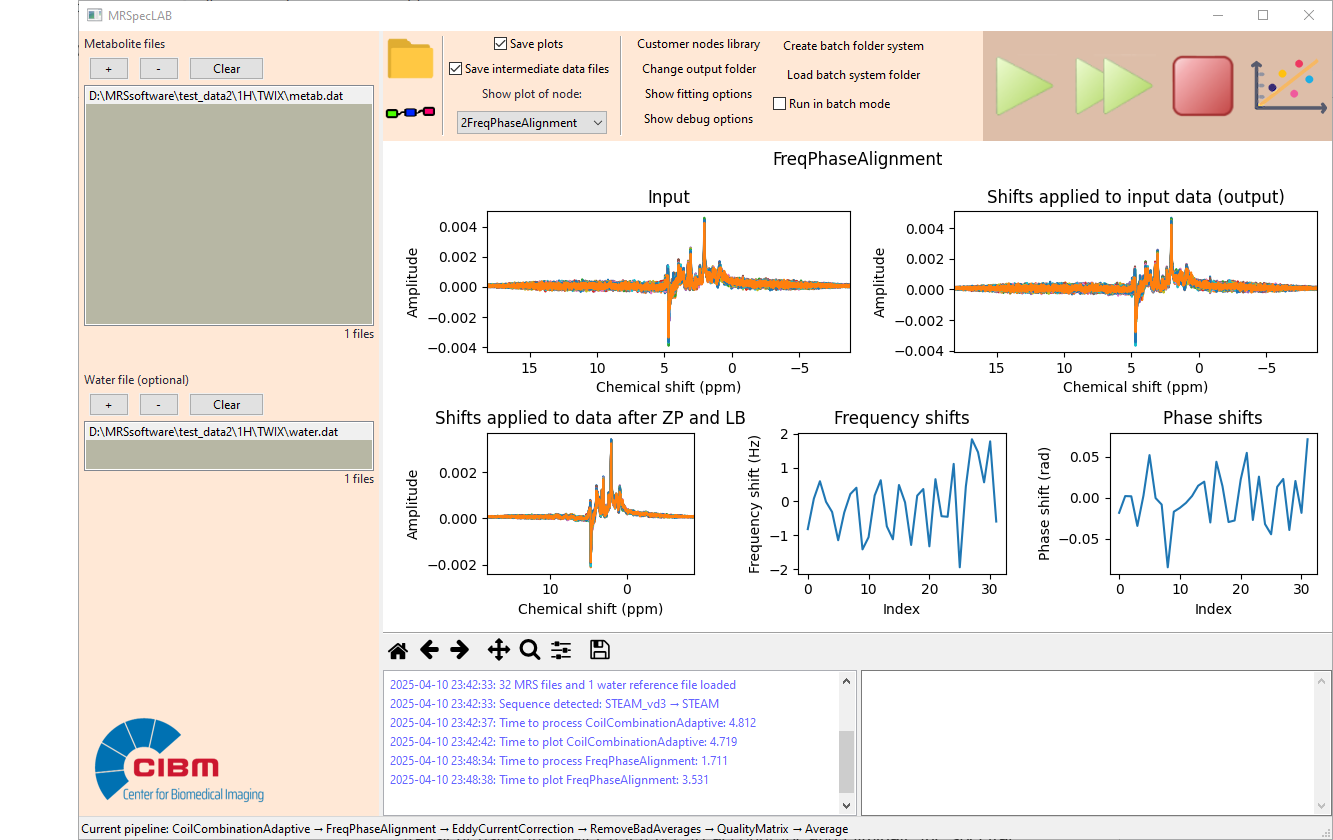
**

1. Eddy current correction: Eddy current correction was performed on each transient using the water reference to account for and eliminate the spectral shape distortions. This step ensures improved spectral accuracy by correcting distortions caused by eddy currents. The non-linear phase before correction and the corrected phase after applying the correction are displayed in the figures.

**
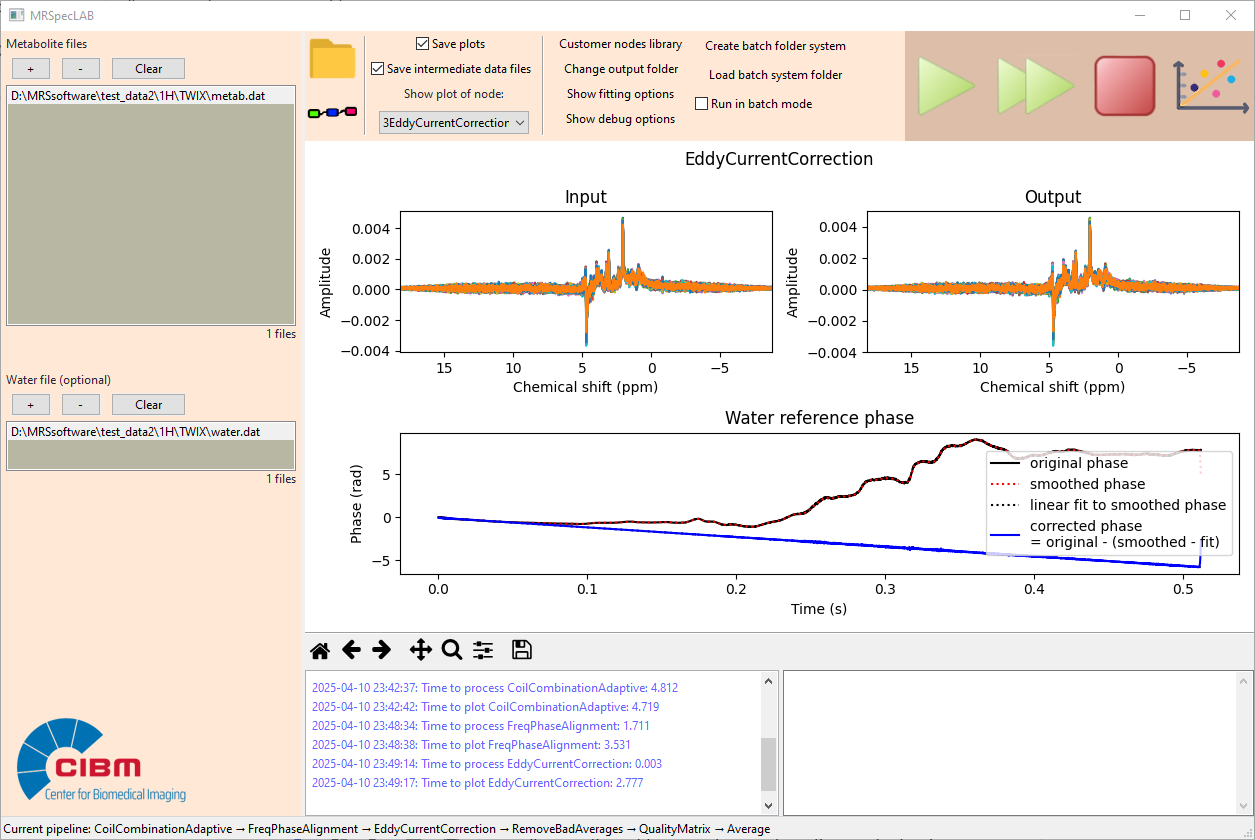
**

1. Bad average removal: Following the eddy current correction, the similarity of each transient is evaluated to detect any deviations from the expected spectral pattern. Outlier transients, which may arise from factors such as hardware or motion, are identified and removed if they do not meet the similarity criteria, ensuring the quality of the dataset. In this particular dataset, no bad averages (shown in red, if any) were detected, so all transients were deemed acceptable (shown in gray) and moved forward to the next step in the processing pipeline.

**
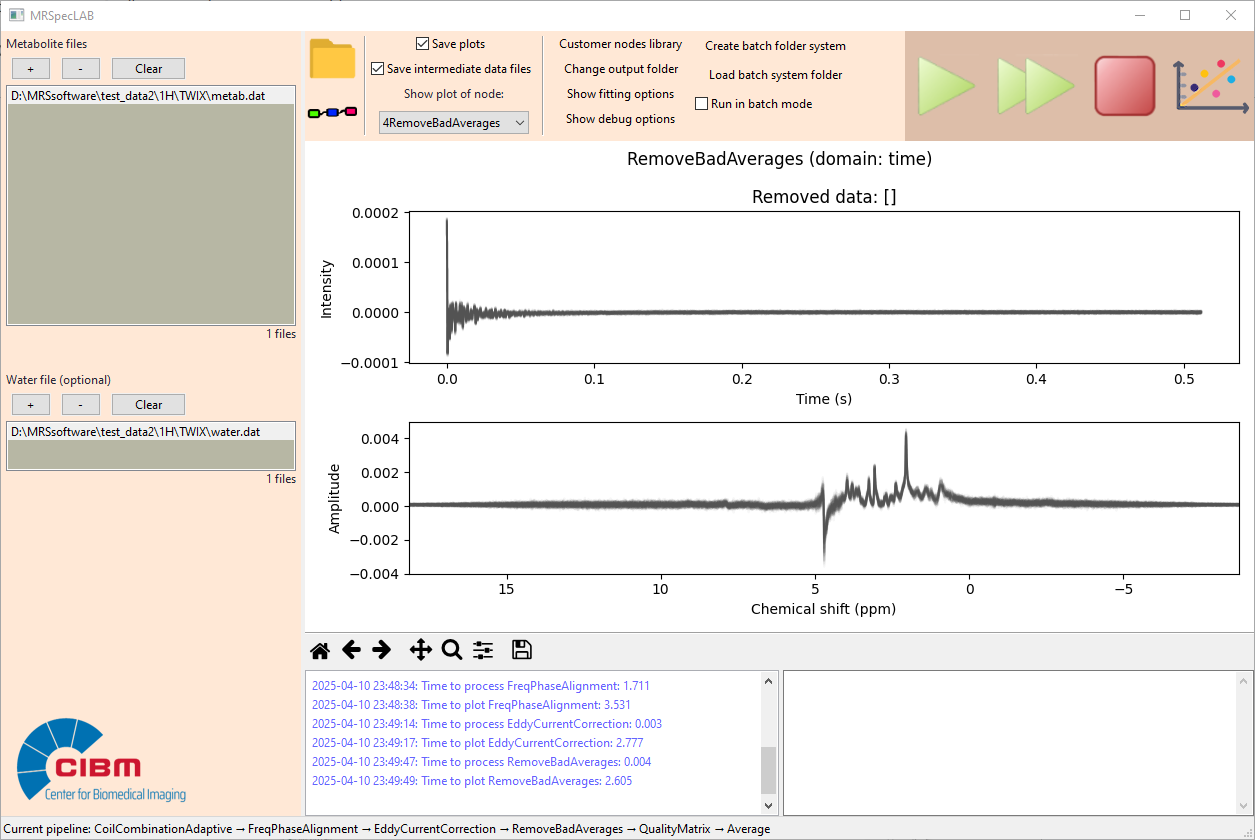
**

1. Quality matrix: assess the quality of processed spectral data by calculating two key metrics: the SNR across the individual transients and the water linewidth. The SNR is computed for each spectrum to evaluate the signal strength of the NAA peak at 2.02 ppm relative to the noise from 0 to 0.5 ppm, indicating data quality. The water linewidth is estimated by fitting a Gaussian function to the water reference spectrum, allowing the calculation of the Full Width at Half Maximum (FWHM).

**
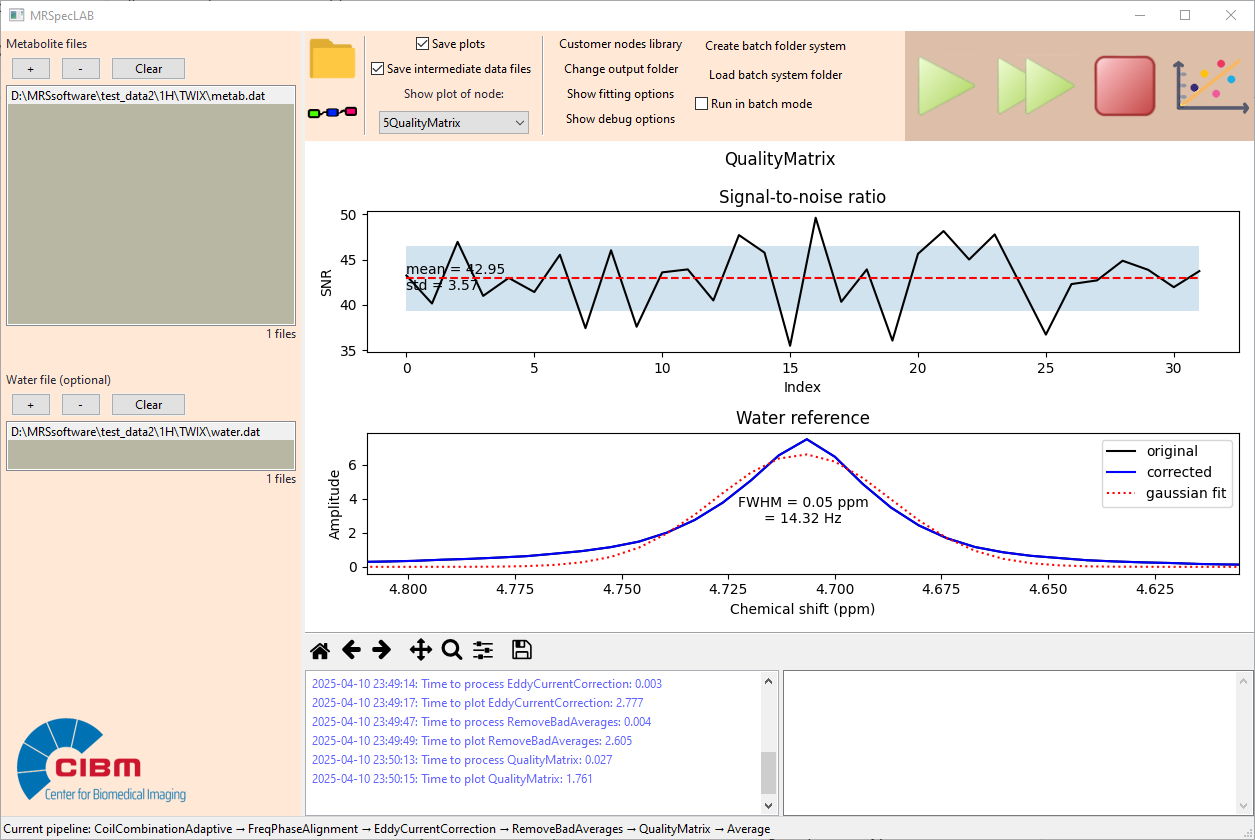
**

1. Spectral averaging: The remaining good transients are averaged together. The left column of the figure shows the time-domain data, while the right column displays the spectra before and after averaging.

**
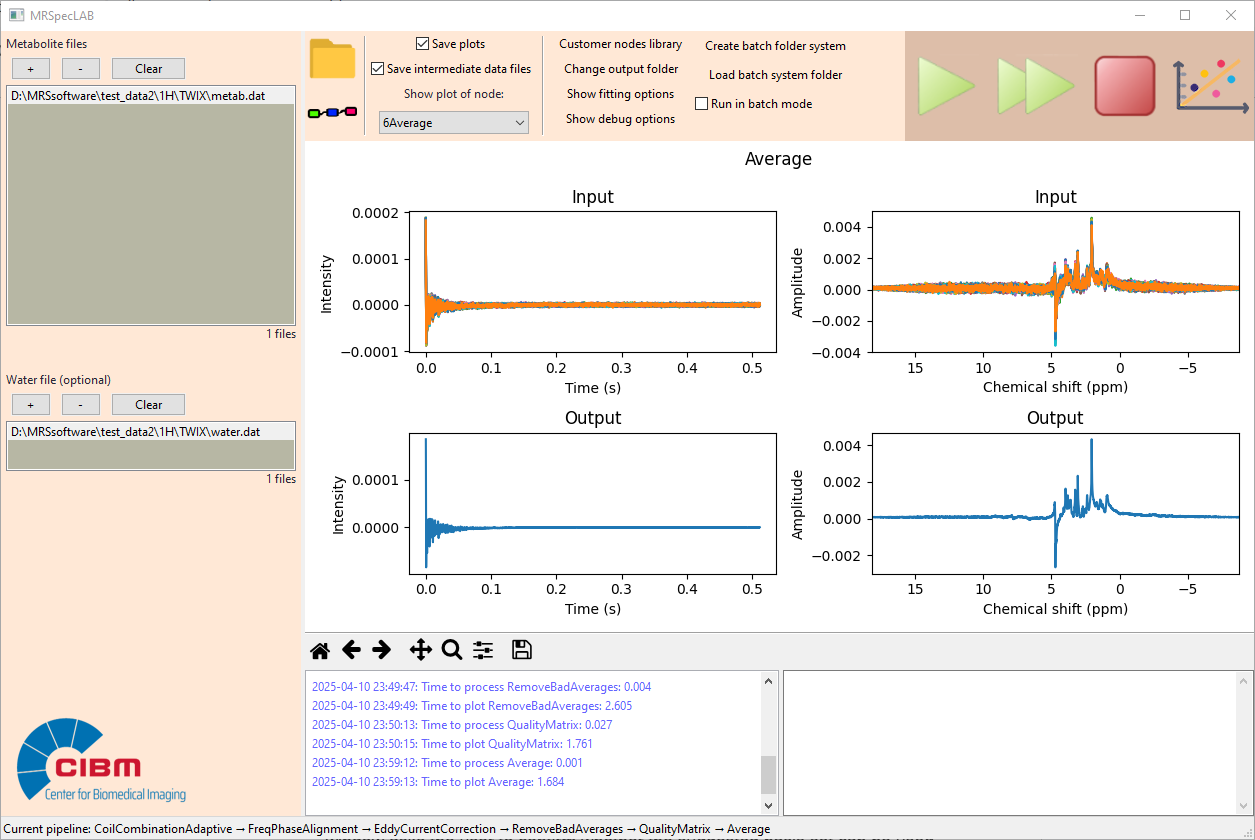
**

1. Spectral fitting: If no specific basis set is selected by the user, MRSpecLAB automatically detects the sequence information, including sequence type and scanning parameters such as TE, and attempts to match the data with a suitable basis set from the default library. Once a basis set is found, a prompt window asks the user to confirm whether the suggested basis set can be used for data fitting. Together with the fitting parameters (specified in the LCModel .control file), the software initiates the LCModel fitting process for the processed data. The final individual spectrum is displayed in the central figure panel, allowing users to visually inspect the fitting results. The quantitative analysis results extracted from the COORD file are summarized in the right information panel, providing users with quick access to key outcomes. Additionally, all fitting results generated by LCModel are systematically organized in the output folder, enabling users to review and utilize the results later as needed.

**
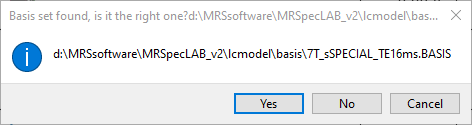
**

**
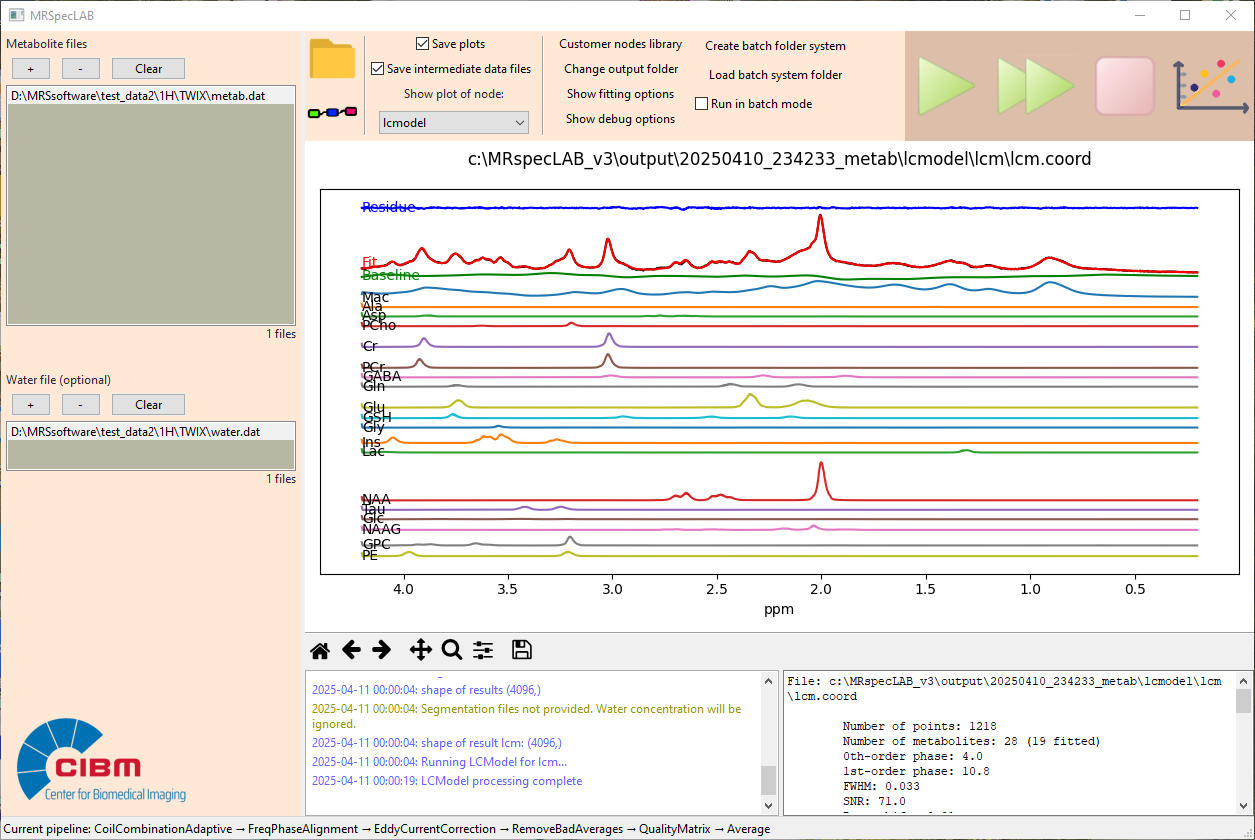
**

# **Supplementary Section 3. Example control file for short-TE svs 1H MR spectral fitting**

MRSpecLAB includes a default .control template for short-TE ^1^H MRS data quantification. Key parameters such as DELTAT, HZPPPM, and NUNFIL, which depend on the sequence and acquisition settings, are automatically extracted from the input data header, so users don’t need to manually adjust these values when analyzing different datasets. Other fitting parameters are predefined in the default .control template and used during the fitting process. If users wish to fit with different parameter configurations, they can create and upload their own .control template through the fitting options before running the analysis. MRSpecLAB will then automatically generate new .control files based on the uploaded template for each individual dataset. Additionally, users do not need to specify the directory or name of the input data in this file, as MRSpecLAB directly links to the processed data, which allows to simplify the workflow and ensure compatibility across various datasets.

$LCMODL

KEY= 210387309

OWNER= 'MetMRS, CIBM, EPFL'

DELTAT= 0.000125

HZPPPM= 297.2041

NUNFIL= 4096

PPMEND= 0.2

PPMST= 4.2

NEACH= 999

LPS= 8

NSIMUL= 0

ECHOT= 4.5

DKNTMN= 0.2

PPMSHF= 0

NUSE1= 4

CHUSE1(1)= 'NAA'

CHUSE1(2)= 'Cr'

CHUSE1(3)= 'Glu'

CHUSE1(4)= 'Ins'

NCOMBI= 4

CHCOMB(1)= 'Glu+Gln'

CHCOMB(2)= 'Cr+PCr'

CHCOMB(3)= 'NAA+NAAG'

CHCOMB(4)= 'GPC+PCho'

VITRO = F

ATTH2O= 1

WCONC= 44444

DEGZER= 0

DOECC = F

DOWS = T

DOREFS = T

SHIFMN = -0.2,-0.1

SHIFMX = 0.3,0.3

$END

# **Supplementary Section 4. Step-by-step processing and visual outputs of ^1^H fMRS data**

fMRS dataset processing pipeline and results

1. Coil combination


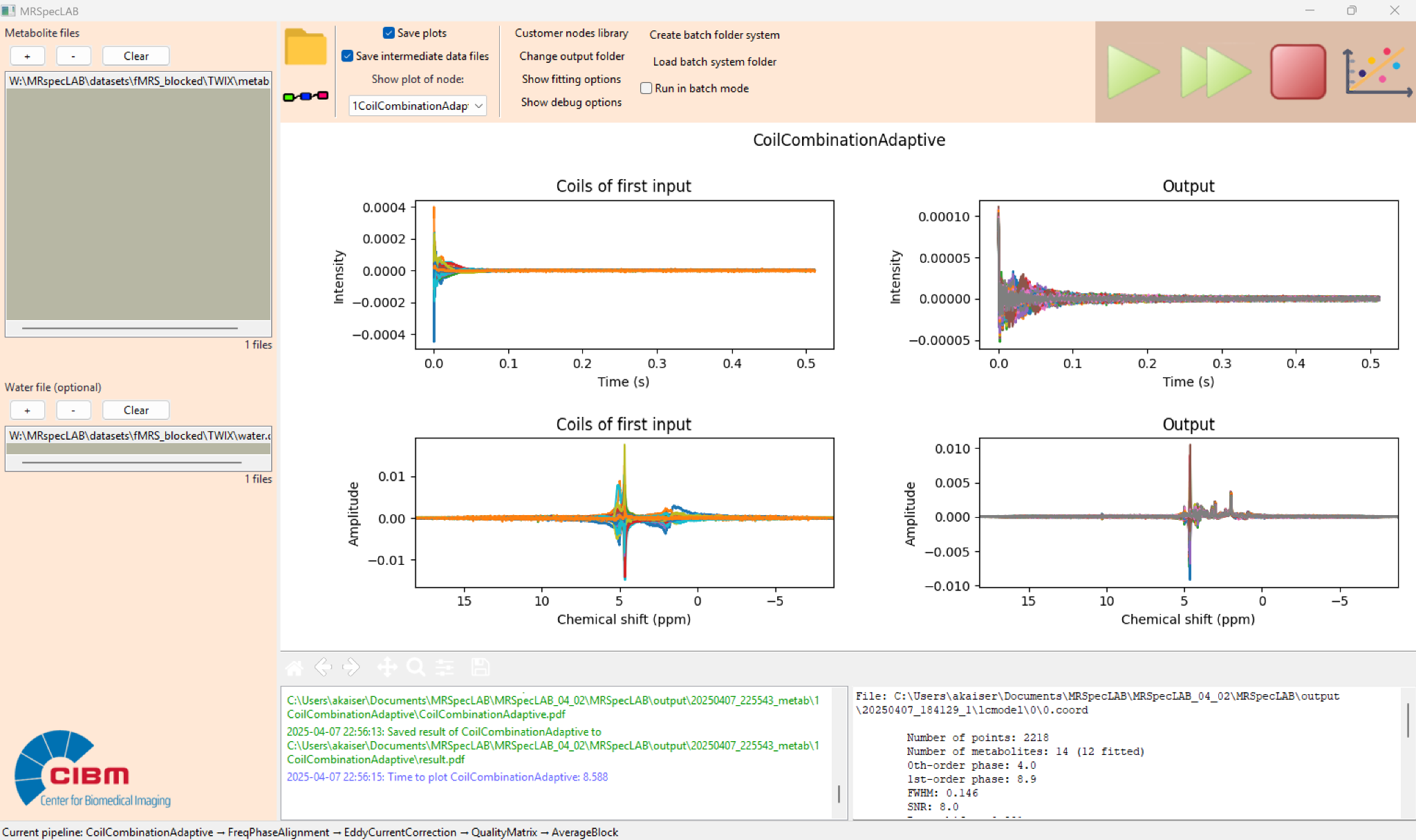


1. Frequency and phase alignment


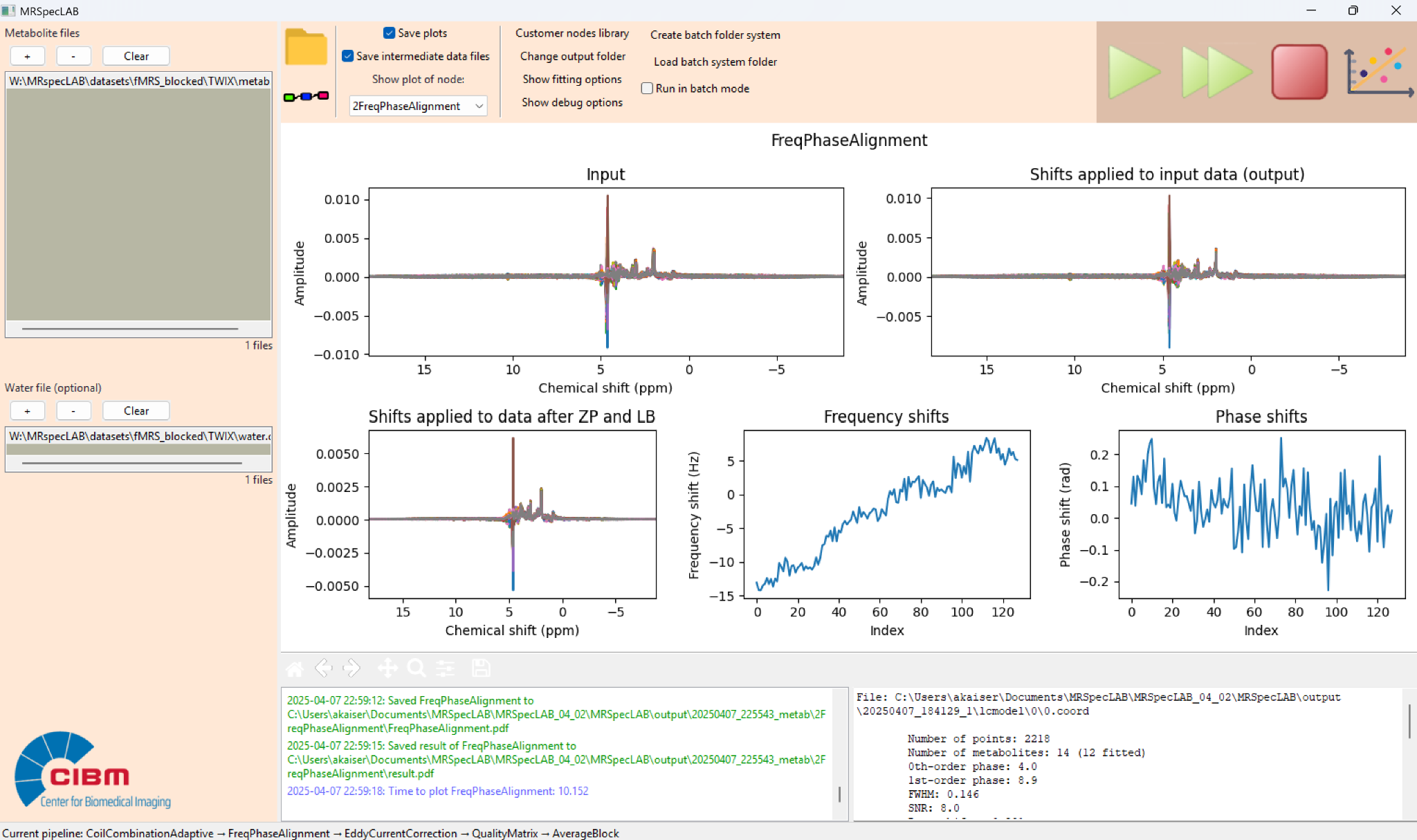


1. Eddy current correction


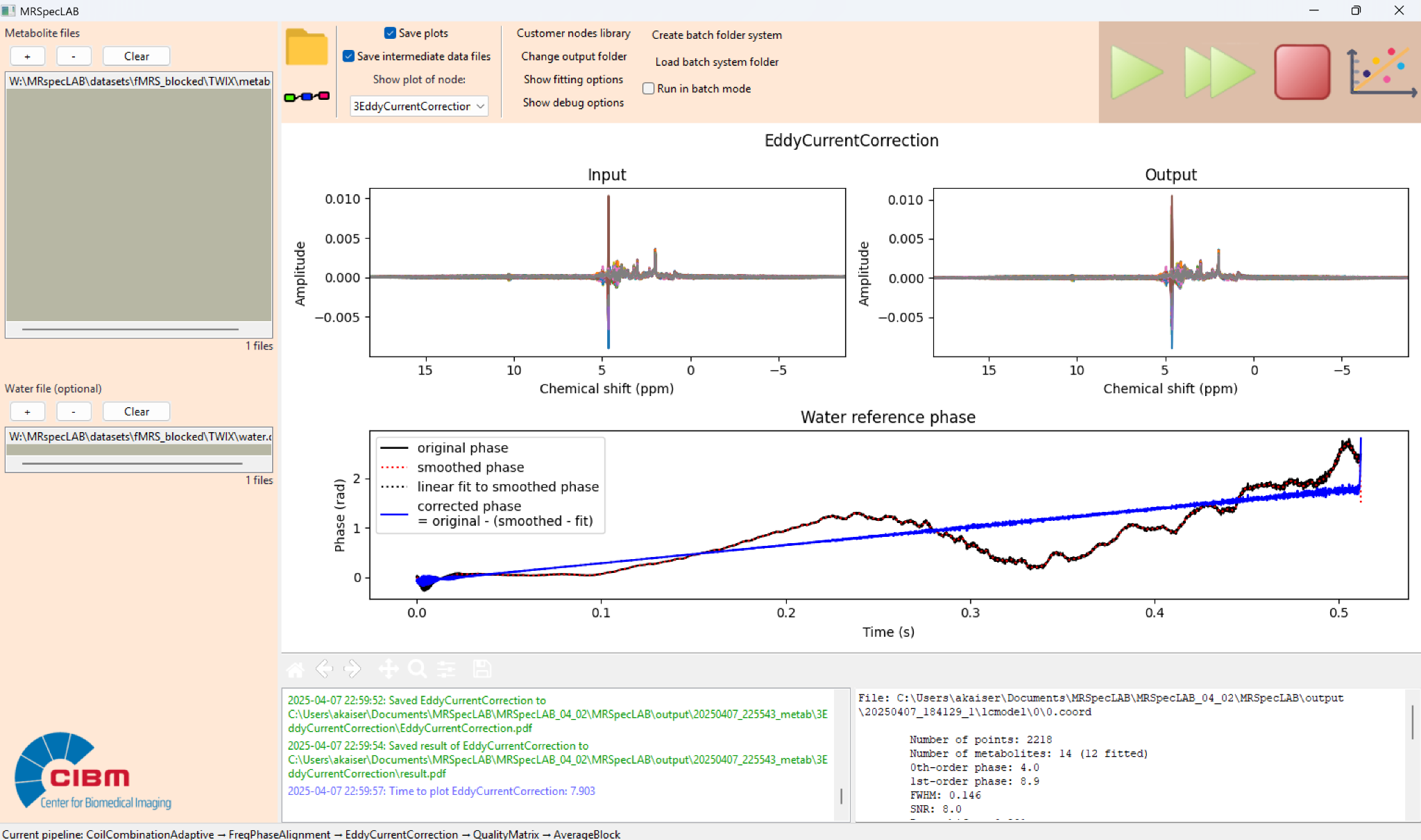


1. Quality matrix outcome


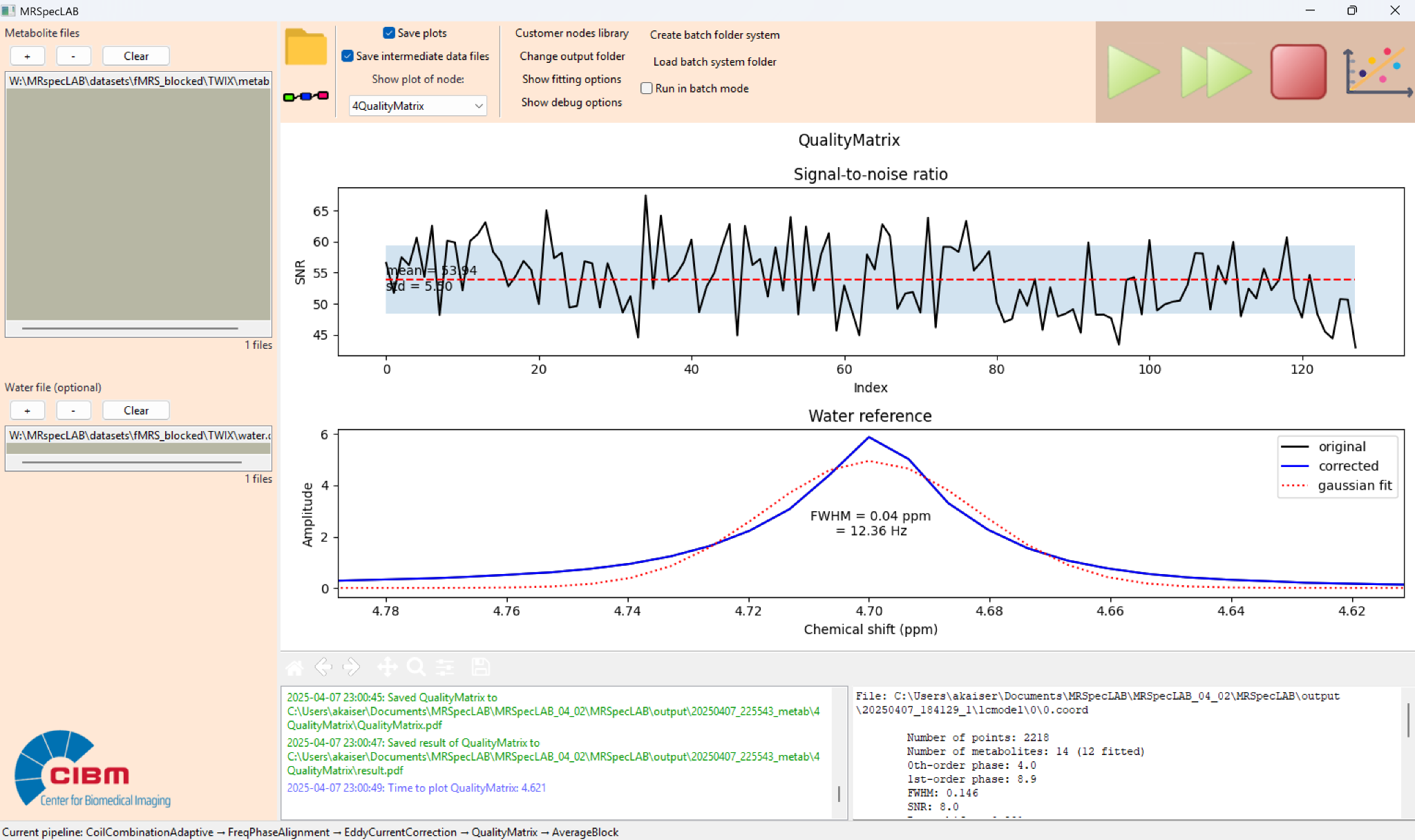


1. Block averaging


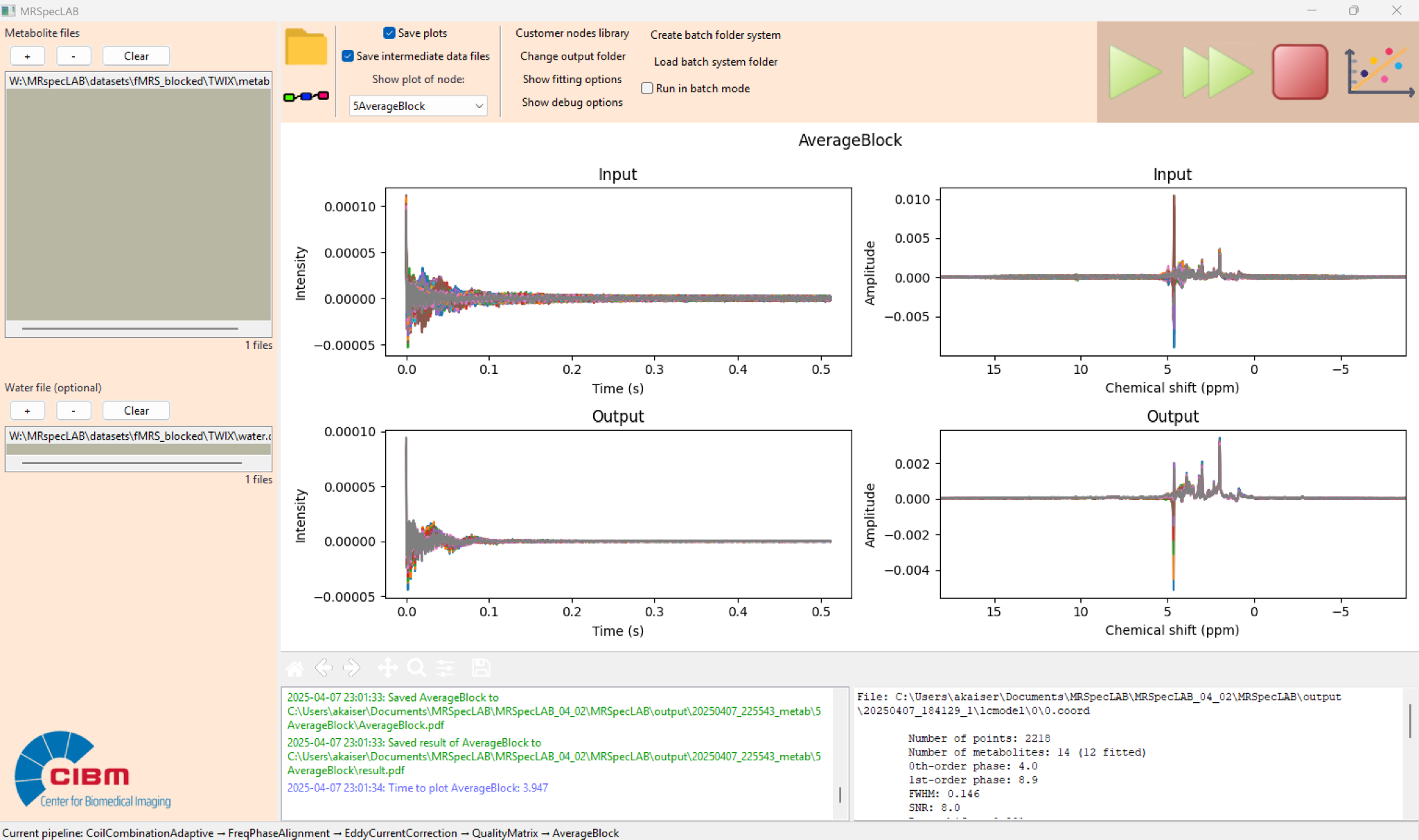


1. LCModel fitting per average


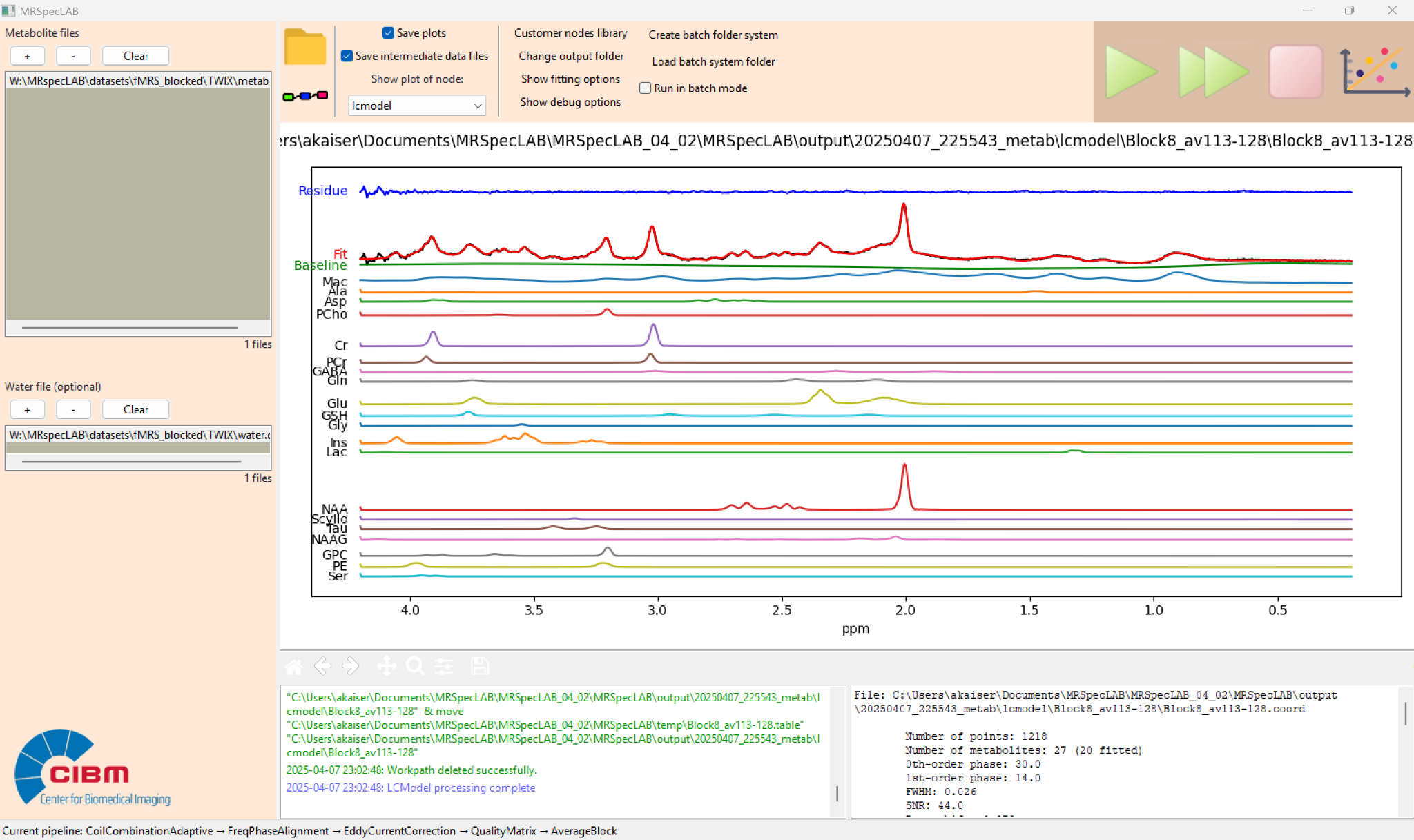


# **Supplementary Section 5. Detailed comparisons of the individual post-processed spectra, and quantification results**

Comparison of post-processed spectra (n=24) using MRSpecLAB and FID-A.

The input data was acquired with and without water suppression in TWIX format (sequence: sSPECIAL, VOI = 25 × 30 × 30 cm^3^, TE/TR = 16/4000 ms, BW = 4 kHz, NT = 16 for water-suppressed data, and NT = 2 for unsuppressed water data).

This analysis provides a comparison of the above mentioned 1H MRS spectra processed with MRSpecLAB and FID-A using identical raw data acquired in Siemens TWIX format. Both analysis pipelines included the same preprocessing steps: coil combination based on the water reference signal, frequency and phase alignment, eddy current correction, outlier average removal, and signal averaging.

For FID-A, the processing pipeline included the following standard functions: io_loadspec_twix → op_addrcvrs → op_alignAverages → op_rmbadaverages → op_averaging → op_ecc.

For MRSpecLAB, the corresponding nodes included were: CoilCombinationAdaptive → FreqPhaseAlignment → EddyCurrentCorrection → RemoveBadAverages → Quality Matrix → Average.

Visual inspection of the processed spectra shows high consistency in spectral features across both methods, with strong similarity in peak shapes, baseline stability, and noise characteristics (Figure below). Five final spectra (each averaged over 16 transients) are shown, with each participant (P1–P5) displayed in a separate row. With participants depicted per row (P1-5) and colors indicating the toolbox used for preprocessing (pink: FID-A, blue: MRSpecLAB).


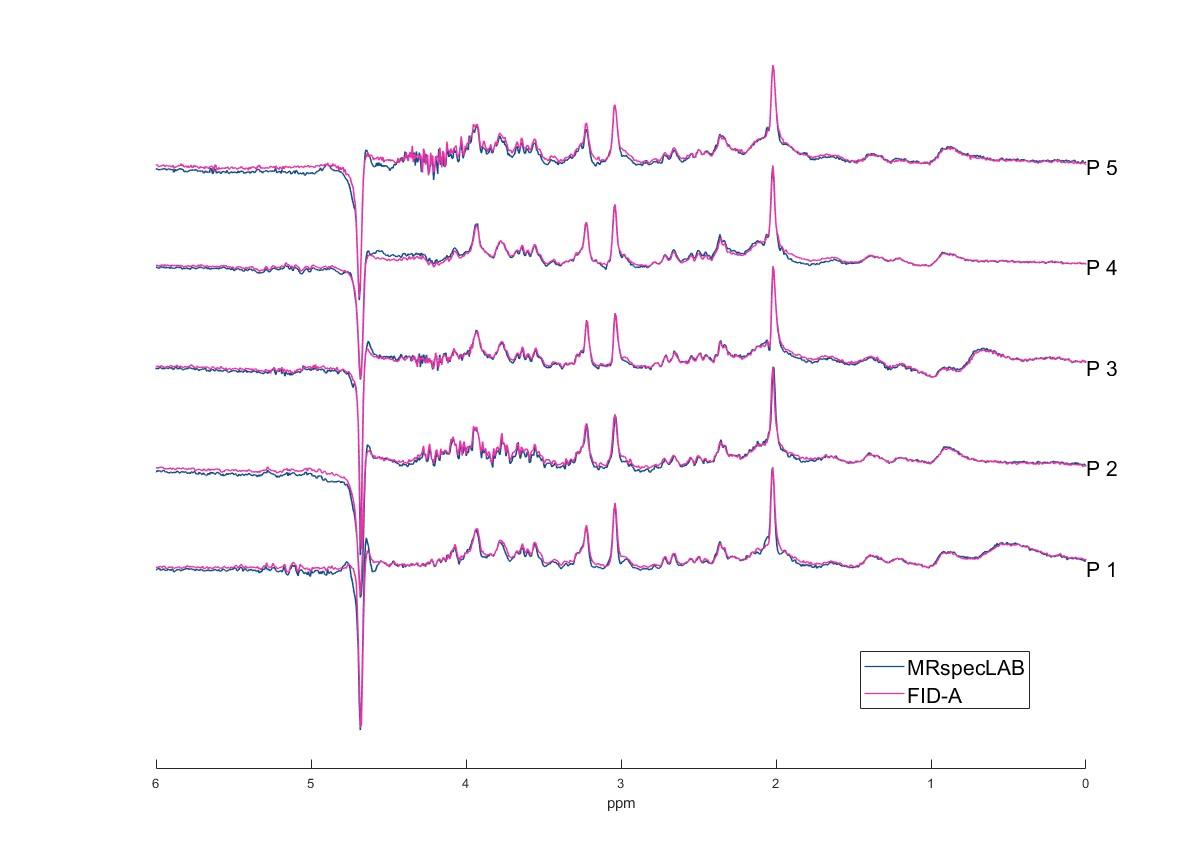


Spectral quantification was performed using LCModel version 6.3 with the same basis set and control file for both toolboxes. The following Bland-Altman plots show the agreement correlation of metabolite concentrations (in mM, no tissue correction, concentration estimation based on assuming water concentration is 44.444 M within the voxel) between MRSpecLAB and FID-A across 24 participants (datasets shown in Figure 5) for tNAA, tCr, Glu. Each point represents an individual participant’s metabolite concentration. The red dashed line represents the mean bias, while the black dashed lines indicate the limits of agreement (±1.96 SD).

**
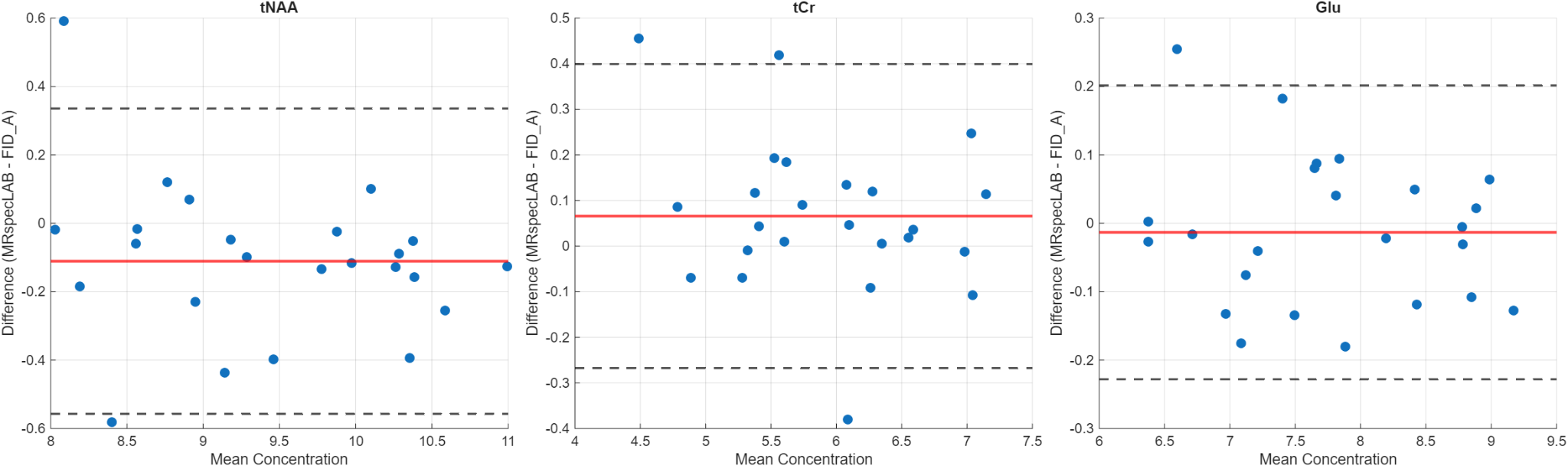
**

The following table compares the metabolite quantification between MRspecLAB and FID-A. It summarizes the agreement between MRspecLAB and FID-A across 3 major metabolites (tNAA, tCr, Glu) using multiple statistical measures. Metrics include the mean bias and 95% limits of agreement (LoA) from Bland–Altman analysis, concordance correlation coefficient (CCC), slope and intercept from Deming regression, and p-values from the Pearson correlation. Bonferroni and FDR-corrected significance values indicate whether the differences are statistically significant after correction for multiple comparisons.

Across all metabolites, small mean biases were observed (|bias| < 0.12 mM) between the two methods, with excellent agreement for tNAA, tCr, and Glu (CCC ≥ 0.96). Deming regression showed slopes near 1 for most high-concentration metabolites. The correlations between the two methods were statistically significant (p < 0.001) and remained significant after Bonferroni and FDR corrections.

| **Metabolite** | **Mean Bias** | **LoA Upper** | **LoA Lower** | **CCC** | **Deming Slope** | **Deming Intercept** | **P-value (Pearson)** | **Bonferroni Significant** | **FDR Significant** |
| --- | --- | --- | --- | --- | --- | --- | --- | --- | --- |
| **tNAA** | **-0.111** | **0.336** | **-0.557** | **0.960** | **0.944** | **0.421** | **< 0.001** | **TRUE** | **TRUE** |
| **tCr** | **0.066** | **0.399** | **-0.268** | **0.970** | **0.945** | **0.392** | **< 0.001** | **TRUE** | **TRUE** |
| **Glu** | **-0.013** | **0.202** | **-0.228** | **0.992** | **0.983** | **0.118** | **< 0.001** | **TRUE** | **TRUE** |

# **Supplementary Section 6. ^31^P dataset processing and results**

1. Apodization of 5 Hz

**
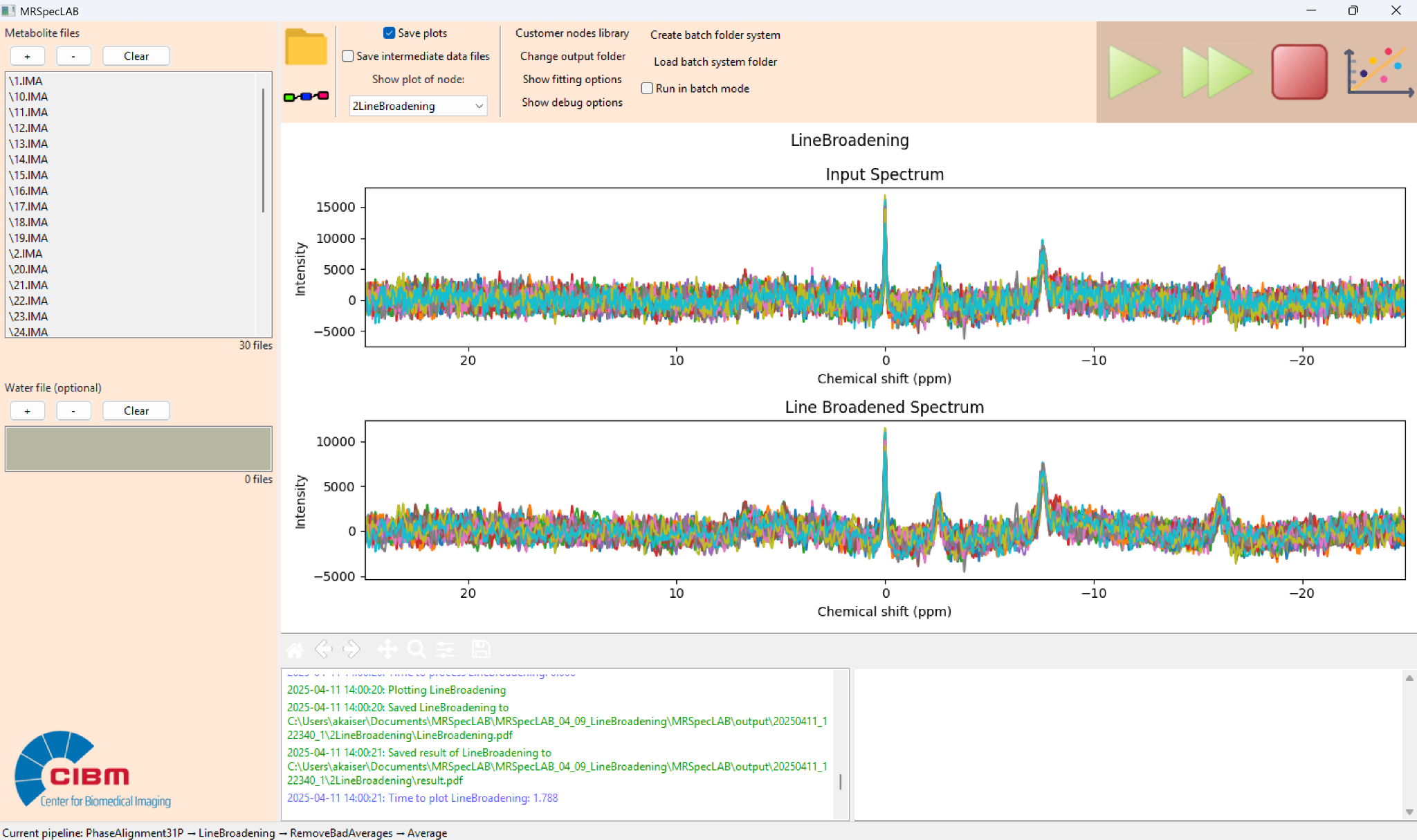
**

1. Averaging

**
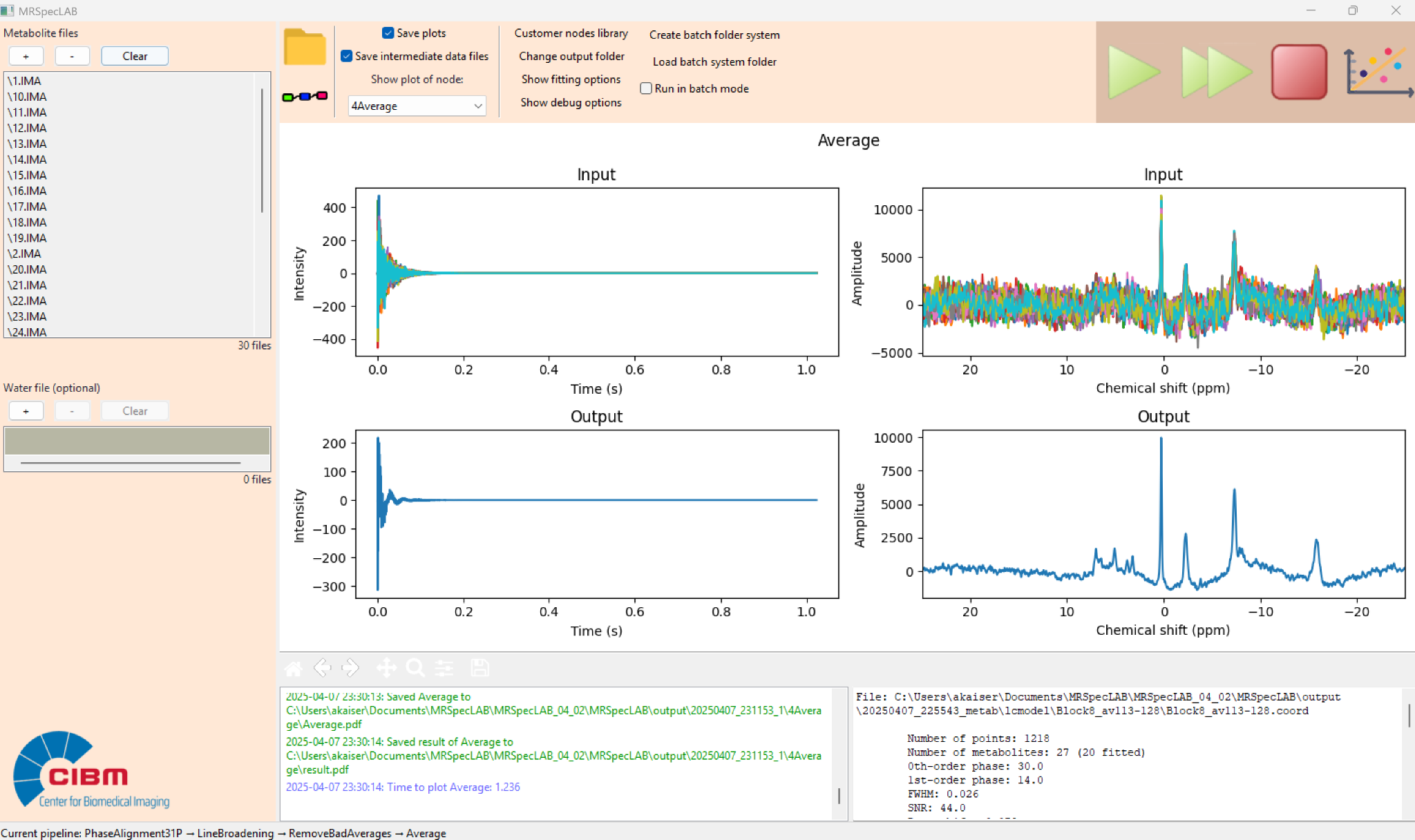
**

1. **LCModel fitting**

**
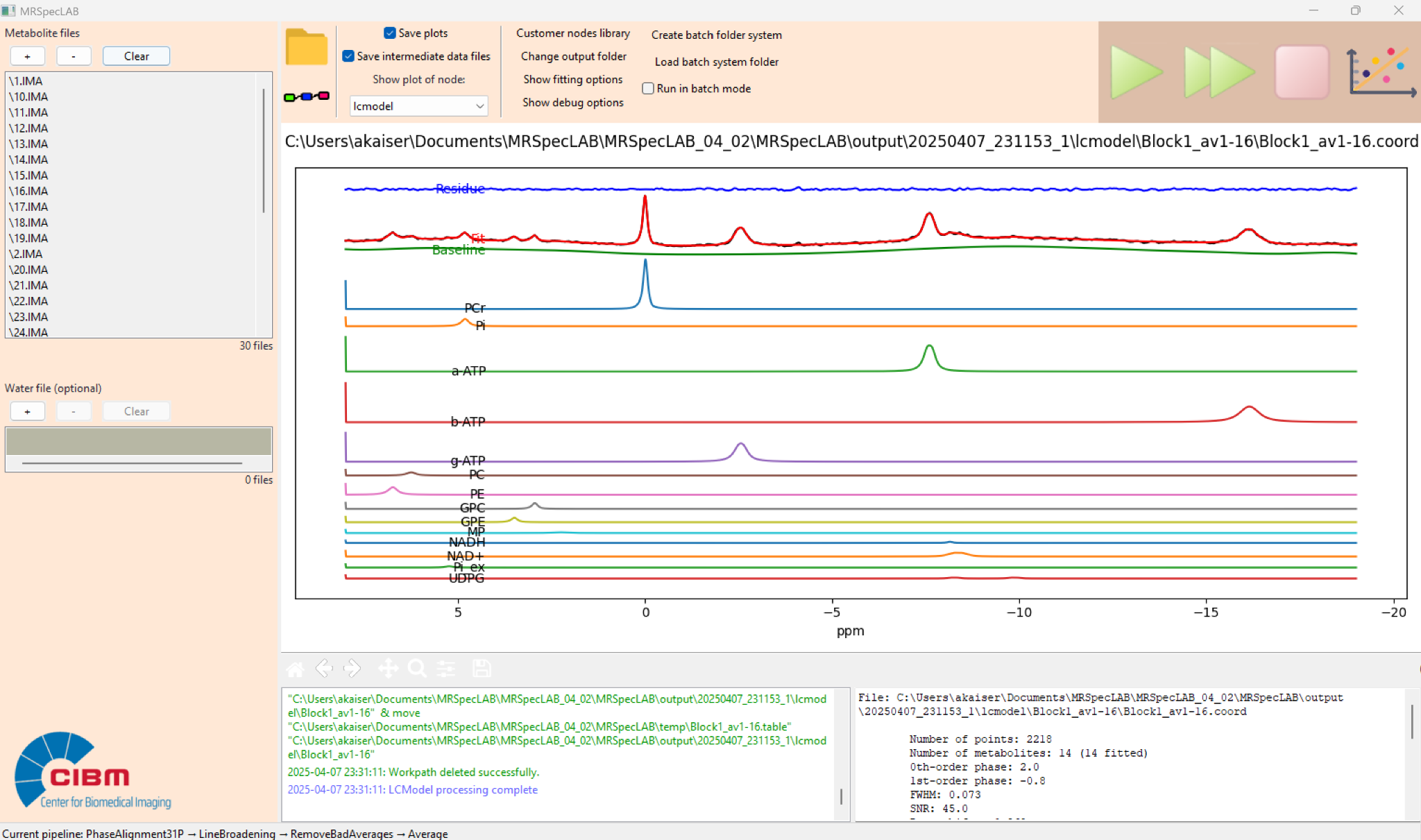
**

# **Supplementary Section 7. ^31^P CSI data processing and fitting**

1. Hanning filter

**
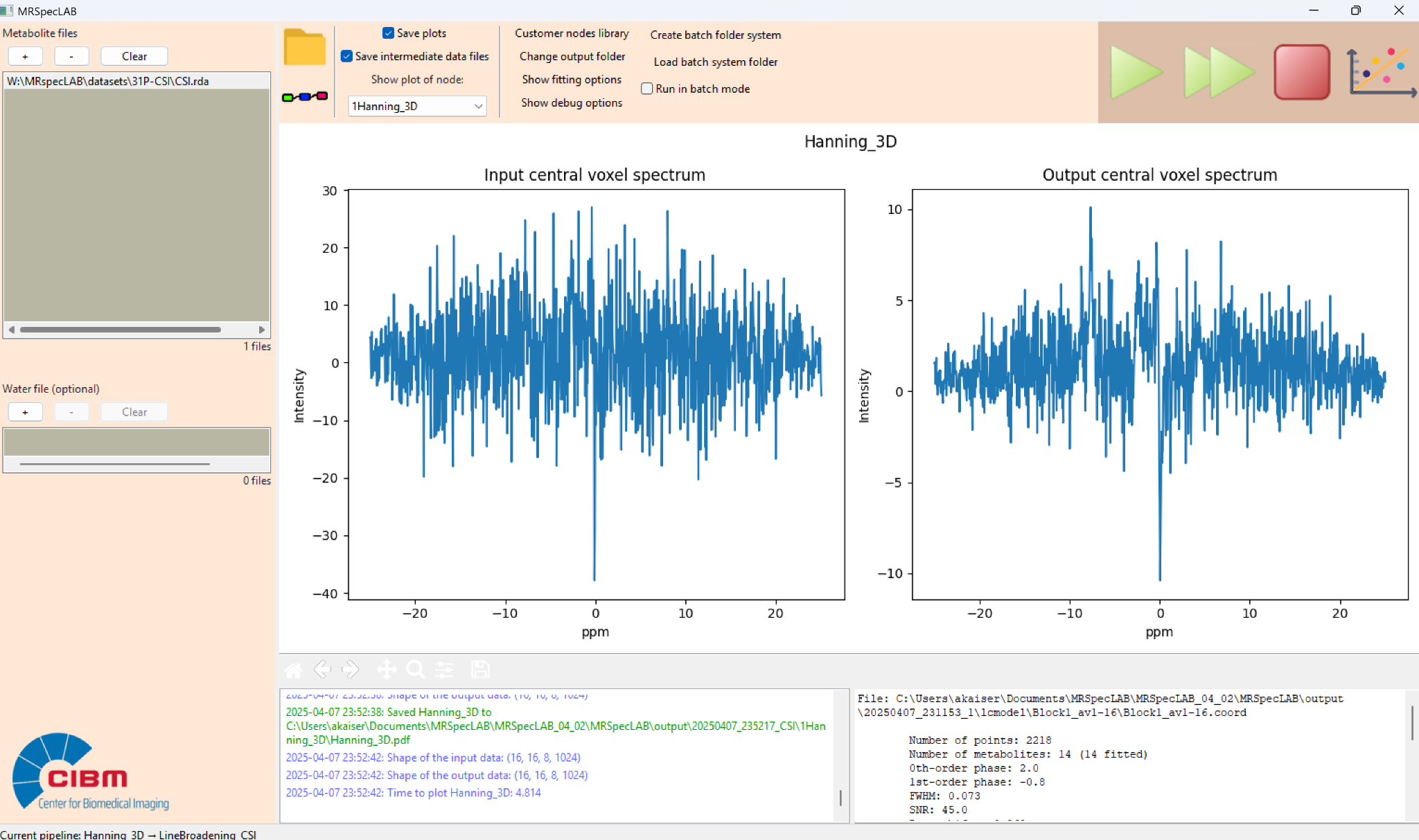
**

1. Apodization 5 Hz

**
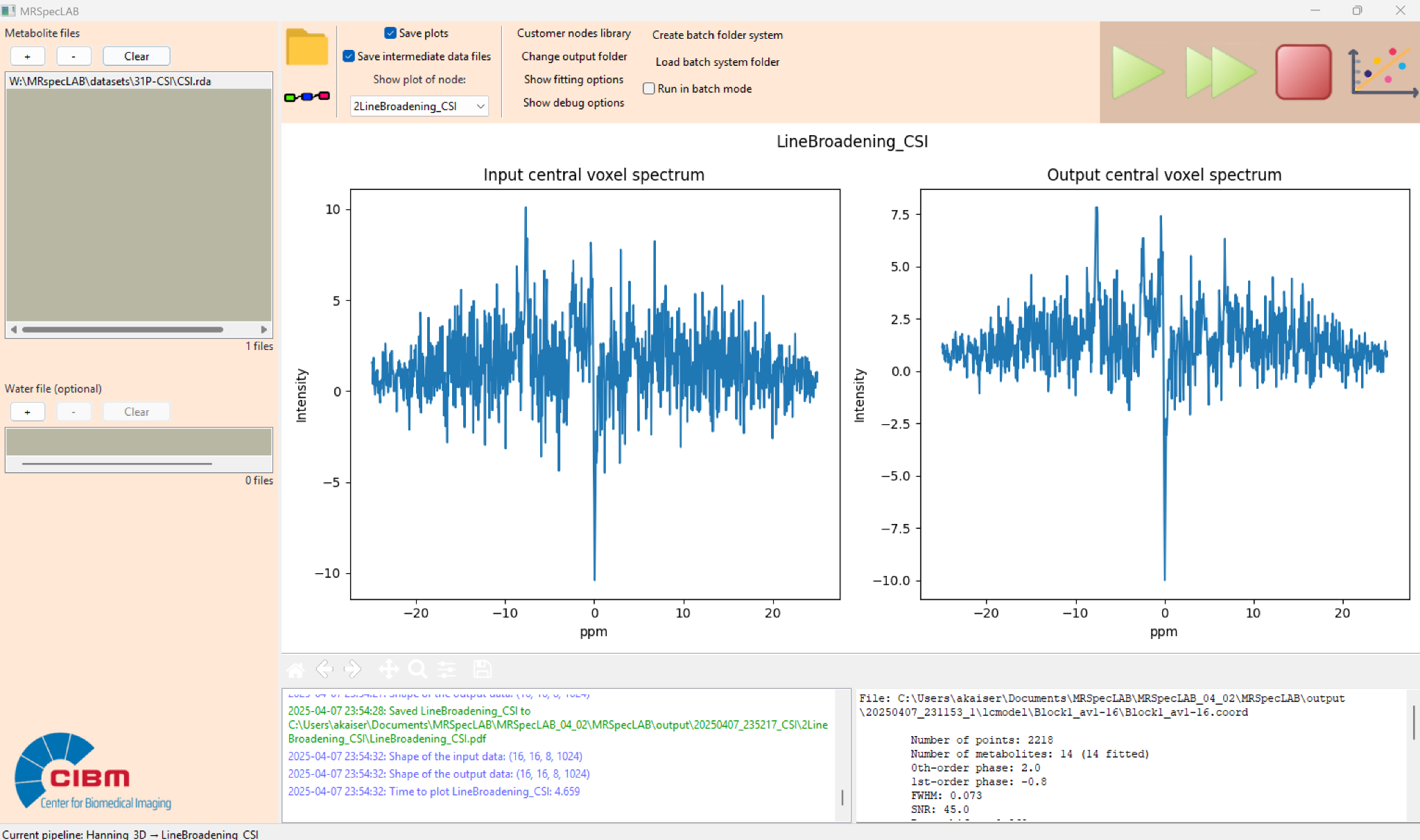
**

1. LCModel fitting (voxel by voxel)

**
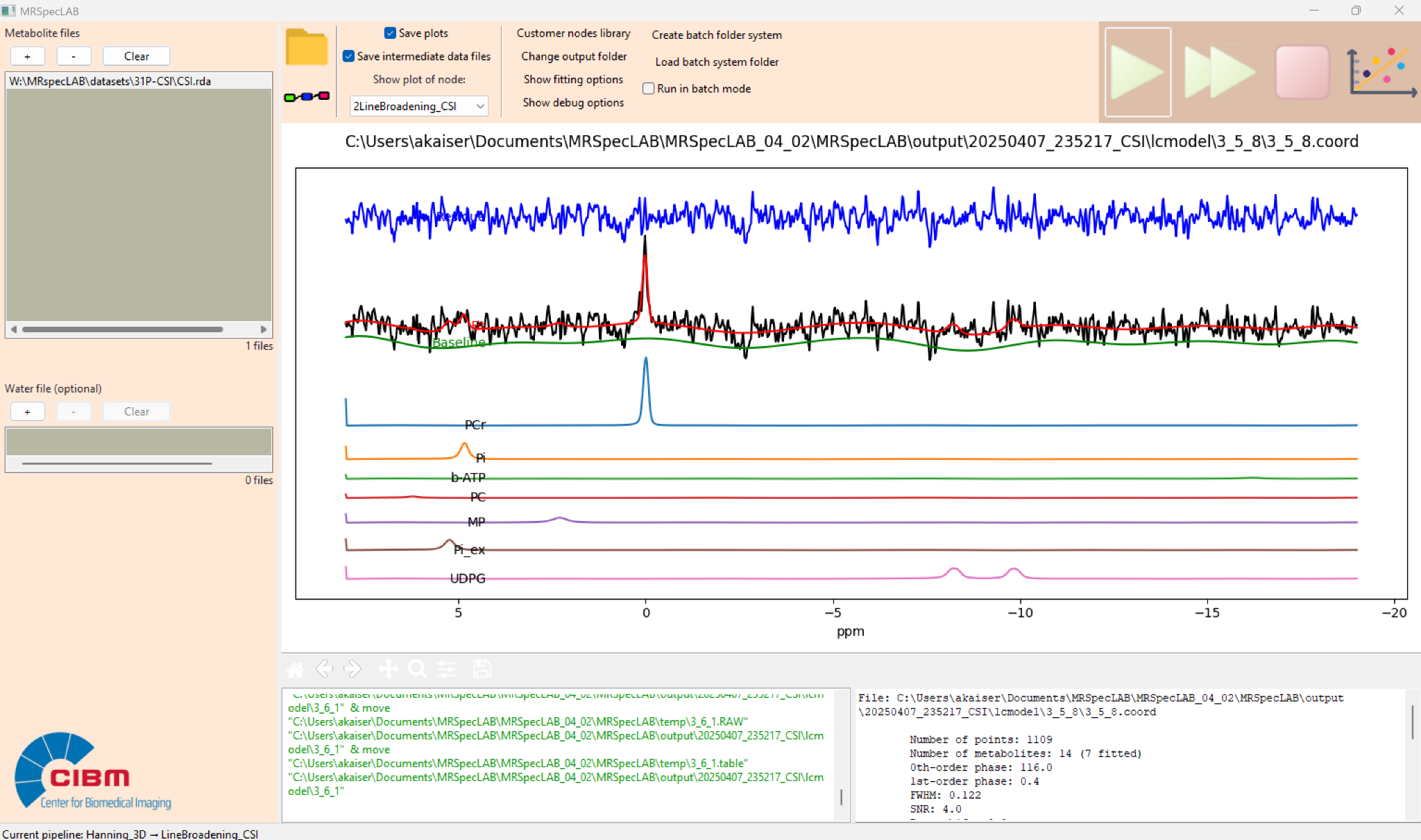
**

# **Supplementary Section 8. Example code for custom node**

Below is an example code for creating a custom node that applies Gaussian line broadening:

# Import necessary APIs and libraries

**import** processing**.**api **as** api

**import** numpy **as** np

# Define the custom node class

**class** **LineBroadening_Gaussian(**api**.**ProcessingNode**):**

**def** __init__**(**self**,** nodegraph**,** **id):**

# Meta information about the node

self**.**meta_info **=** **{**

"label"**:** "Line Broadening (Gaussian)"**,**

"author"**:** "MetMRS"**,**

"description"**:** "Applies Gaussian line broadening to spectral data"**,**

**}**

# Define adjustable parameters

self**.**parameters **=** **[**

api**.**IntegerProp**(**

idname**=**"Gaussian_lw_hz"**,**

default**=**5**,** # Default linewidth (Hz)

min_val**=**1**,** # Minimum value

max_val**=**50**,** # Maximum value

fpb_label**=**"Linewidth (Hz)" # Label for the GUI

**)**

**]**

**super().**__init__**(**nodegraph**,** **id)** # Initialize the parent class

self**.**plotSpectrum **=** **False** # Disable spectrum plotting by default

**def** process**(**self**,** data**):**

# Create Gaussian apodization function

self**.**exp **=** np**.**exp**(-((**data**[**"input"**][**0**].**time_axis**()** ***** np**.**pi *****

self**.**get_parameter**(**"Gaussian_lw_hz"**))** **/**

**(**2 ***** np**.**sqrt**(**np**.**log**(**2**))))** ****** 2**)**

output **=** **[]**

self**.**dmax **=** 0

**for** d **in** data**[**"input"**]:**

# Apply the defined function

output**.**append**(**d**.**inherit**(**d ***** self**.**exp**))**

self**.**dmax **=** **max(**self**.**dmax**,** np**.max(**d**))** # Track max value for plotting

data**[**"output"**]** **=** output

**def** plot**(**self**,** figure**,** data**):**

# Plot input, apodization function, and output

figure**.**suptitle**(**self**.**__class__**.**__name__**)**

ax **=** figure**.**add_subplot**(**2**,** 1**,** 1**)**

**for** d **in** data**[**"input"**]:**

ax**.**plot**(**d**.**time_axis**(),** np**.**real**(**d**))**

ax**.**plot**(**d**.**time_axis**(),** self**.**exp ***** self**.**dmax**,** ':k'**)**

ax**.**set_xlabel**(**'Time (s)'**)**

ax**.**set_ylabel**(**'Signal Intensity (a.u.)'**)**

ax**.**set_title**(**"Input and Apodization Function"**)**

ax **=** figure**.**add_subplot**(**2**,** 1**,** 2**)**

**for** d **in** data**[**"output"**]:**

ax**.**plot**(**d**.**time_axis**(),** np**.**real**(**d**))**

ax**.**set_xlabel**(**'Time (s)'**)**

ax**.**set_ylabel**(**'Signal Intensity (a.u.)'**)**

ax**.**set_title**(**"Output"**)**

figure**.**tight_layout**()**

# Register the custom node to make it available in the node library

api**.**RegisterNode**(**LineBroadening_Gaussian**,** "LineBroadening_Gaussian"**)**

# **Supplementary Section 9. Output data structure**

Output is organized by numbered folders per processing step. Each folder contains a diagnostic plot for quality control of the respective step, and a results figure showing the resulting spectra (.PDF). Additionally, if selected, the results of every processing step are saved in a ‘’data’’ folder as LCModel .RAW output and NIFTI-MRS. The LCModel output folder contains several files: .control: Contains all setting parameters for the fit; .coord: Includes fitted spectral curves, quality control parameters, and final fit results; .csv: Summarizes quantification results per metabolite; .ps: Provides a visual representation of all fitted spectra; .print: Contains detailed analysis information, including metabolite concentrations and correlations; .nii: Stores fitted metabolite maps in NIfTI format; .H2O: Contains raw water spectral data; .table: Presents quantification results in tabulated format; .RAW: Stores raw metabolite spectral data in the time domain (Provencher SW. NMR Biomed. 2001;14(4):260–4.).

OUTPUT_FOLDER/

├── pipeline.pipe # Saves the pipeline settings used for processing

├── MRSinMRS_table.csv # Standardized table for MRS acquisition and processing parameters

├── 1CoilCombination/ # First processing step (Coil Combination)

│ ├── data/

│ │ ├── <FILENAME>.RAW # Raw metabolite spectral data (ASCII format, if selected)

│ │ ├── <FILENAME>.nii # Intermediate results in NIfTI-MRS format (if selected)

│ ├── results.pdf # Results of Coil Combination processing

│ ├── CoilCombination.pdf # Quality control output for Coil Combination

├── 2Preprocessing/ # Second processing step (Preprocessing)

│ ├── data/

│ │ ├── <FILENAME>.RAW # Processed spectral data (if selected)

│ │ ├── <FILENAME>.nii # Processed spectral data in NIfTI format (if selected)

│ ├── results.PDF # Results of Preprocessing

│ ├── Preprocessing.pdf # Quality control output for Preprocessing

├── 3Averaging/ # Third processing step (Averaging)

│ ├── data/

│ │ ├── <FILENAME>.RAW # Averaged spectral data (if selected)

│ │ ├── <FILENAME>.nii # Averaged spectral data in NIfTI format (if selected)

│ ├── results.PDF # Results of Averaging

│ ├── Averaging.pdf # Quality control output for Averaging

├── LCModel/ # LCModel output folder (at the same level as processing steps)

│ ├── <FILENAME>.control # LCModel control file with all fit settings

│ ├── <FILENAME>.basis # Basis set used for spectral fitting

│ ├── <FILENAME>.coord # Fitted spectral curves and quality control parameters

│ ├── <FILENAME>.csv # Quantification results per metabolite

│ ├── <FILENAME>.ps # Visual representation of all fitted spectra

│ ├── <FILENAME>.print #Detailed analysis output including metabolite concentrations

│ ├── <FILENAME>.nii # Fitted metabolite maps in NIfTI format

│ ├── <FILENAME>.table # Quantification results in tabulated format

│ ├── <FILENAME>.H2O # Raw water spectral data for quantification (optional)

│ ├── <FILENAME>.RAW # Raw metabolite spectral data in the time domain

**Example output folder in Application 1:**

The data and figures from each step are organized in subfolders named after the corresponding processing nodes. A summary of the data information is provided in the MRSinMRS table. Additionally, the processing pipeline is stored within the output folder.

**
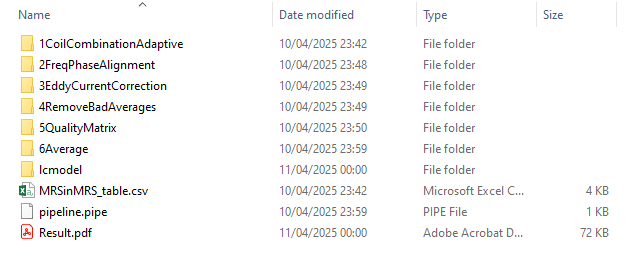
**

For example, in 1CoilCombinationAdaptive, we will have:

**
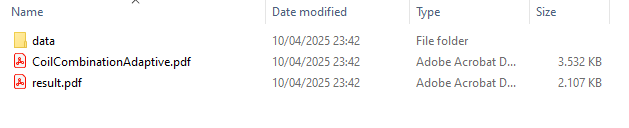
**Once "Save plots" is clicked, all time and frequency domain figures will be saved in PDF format under each step's folder, named "results.pdf" for the user's review. Additionally, a PDF with the same name as the processing node will be saved, containing the figure currently displayed on the main canvas.

**
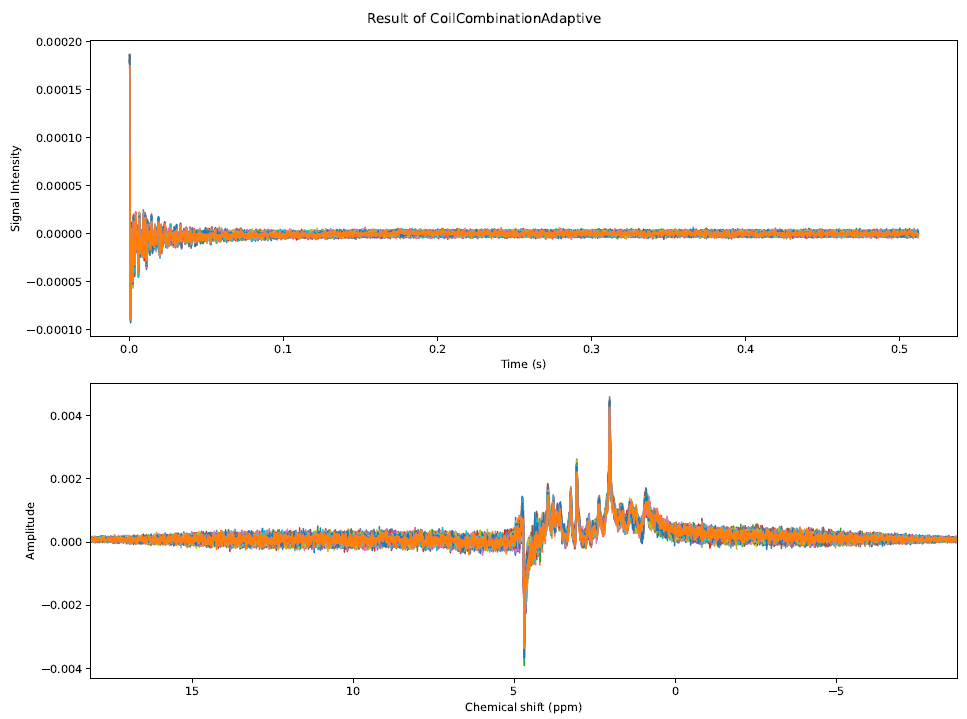
**

Once "Save intermediate datafiles" is clicked, all intermediate time domain data will be stored in .RAW and .nii formats. These files can be found in the subfolder named "data" within each processing step's folder.

**
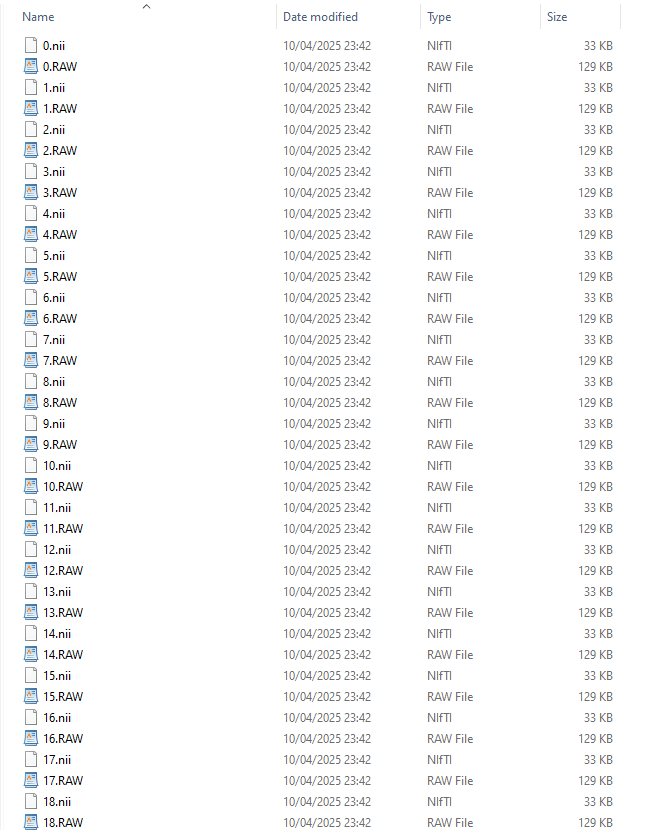
**
